# Liver stage malaria infection is controlled by host regulators of lipid peroxidation

**DOI:** 10.1038/s41418-019-0338-1

**Published:** 2019-05-07

**Authors:** Heather S. Kain, Elizabeth K. K. Glennon, Kamalakannan Vijayan, Nadia Arang, Alyse N. Douglass, Chelsea L. Fortin, Meghan Zuck, Adam J. Lewis, Samantha L. Whiteside, Denali R. Dudgeon, Jarrod S. Johnson, Alan Aderem, Kelly R. Stevens, Alexis Kaushansky

**Affiliations:** 1 Center for Infectious Disease Research, Seattle Biomedical Research Institute, Seattle, WA USA; 2 Seattle Children’s Research Institute, Seattle, WA USA; 3 Departments of Bioengineering & Pathology, Institute for Stem Cell and Regenerative Medicine, University of Washington, Seattle, WA USA; 4 Department of Immunology, University of Washington, Seattle, WA USA; 5 Department of Global Health, University of Washington, Seattle, WA USA; 6 Present Address: Department of Biomedical Sciences, University of California, San Diego, La Jolla, CA USA; 7 Present Address: Pathobiology Program, University of Washington, Seattle, WA USA; 8 Present Address: Department of Biochemistry, University of Utah, Salt Lake City, UT USA

**Keywords:** Infectious diseases, Microbiology

## Abstract

The facets of host control during *Plasmodium* liver infection remain largely unknown. We find that the SLC7a11-GPX4 pathway, which has been associated with the production of reactive oxygen species, lipid peroxidation, and a form of cell death called ferroptosis, plays a critical role in control of *Plasmodium* liver stage infection. Specifically, blocking GPX4 or SLC7a11 dramatically reduces *Plasmodium* liver stage parasite infection. In contrast, blocking negative regulators of this pathway, NOX1 and TFR1, leads to an increase in liver stage infection. We have shown previously that increased levels of P53 reduces *Plasmodium* LS burden in an apoptosis-independent manner. Here, we demonstrate that increased P53 is unable to control parasite burden during NOX1 or TFR1 knockdown, or in the presence of ROS scavenging or when lipid peroxidation is blocked. Additionally, SLC7a11 inhibitors Erastin and Sorafenib reduce infection. Thus, blocking the host SLC7a11-GPX4 pathway serves to selectively elevate lipid peroxides in infected cells, which localize within the parasite and lead to the elimination of liver stage parasites.

## Introduction

*Plasmodium* parasites, the causative agents of malaria, are first transmitted to mammalian hosts by infected *Anopheles* mosquitos. After transmission, parasites travel rapidly through the bloodstream to the liver, where each parasite infects a hepatocyte to form a liver stage (LS) parasite [1, 2]. Only after the completion of LS infection do malaria parasites exit the liver, re-enter the bloodstream, infect erythrocytes, and initiate symptomatic malaria. Previous literature highlights the importance of host cell variation, which can drastically alter susceptibility to infection. In one study using the rodent malaria species *Plasmodium berghei*, the number of sporozoites required to initiate blood stage infection varied from 50 to 10,000 across different strains of naive mice [3]. We have demonstrated that some differences in infectivity originate from differences in hepatocyte biology [4], suggesting that variation in the host can drastically alter susceptibility to infection.

Conventional innate immune defense mechanisms appear to play only a modest role in curtailing initial infection during the liver stage of malaria. Blocking caspase-dependent death in hepatocytes increases LS burden by less than two-fold [5]. The elimination of Toll-like receptor (TLR) 3, TLR4 and the type I interferon receptor have little to no impact on initial LS burden [6, 7], and the elimination of TLR2 modestly increases liver stage infection [8]. Previous work has demonstrated that heme oxygenase (HO), an enzyme that promotes the metabolism of free heme and reduces reactive oxygen species (ROS) is critical for the promotion of LS infection [9]. Moreover, an iron-deficient diet leads to an increase in LS burden [10], and ROS generated by fatty acid β-oxidation impacts *Plasmodium* survival inside hepatocytes [11]. This suggests that other host-driven signaling cascades that promote ROS may contribute to the control of infection.

We have previously shown that infected hepatocytes exhibit diminished levels of P53, and reversing this phenomenon using a small molecule agonist, or with additional genomic copies of P53, reduces liver stage burden [12]. Interestingly, this effect is not based on the capacity of P53 to induce apoptosis [13]. Recent evidence has suggested that P53’s canonical roles in promoting apoptosis, cell cycle arrest and senescence can be dispensable for P53’s capacity as a tumor suppressor [14]. Specifically, a mutant of P53 acts as a potent tumor suppressor by blocking the activity of SLC7a11, a cysteine/glutamate antiporter, and inducing a form of cell death called ferroptosis, which is dependent on the production and accumulation of ROS and the resultant lipid peroxidation [15, 16]. Here, we investigate the role of the SLC7a11 pathway in regulating liver stage malaria infection.

## Materials and Methods

### Cell lines and culture

Hepa1–6 Cells were obtained from ATCC. 293FT cells were obtained from Invitrogen. Cells were maintained in Dulbecco’s Modified Eagle Medium (DMEM) complete media (Cellgro, Manassas, VA, USA), supplemented with 10% FBS (Sigma-Aldrich, St. Louis, MO, USA), 100 IU/ml penicillin (Cellgro), 100 mg/ml streptomycin (Cellgro), 2.5 mg/ml fungizone (HyClone/Thermo Fisher, Waltham, MA, USA), and 5 mg/ml gentamicin (BioWhittaker/Lonza, Basel, Switzerland), and split 1–2 times weekly. Where indicated, cells were treated with Nutlin-3 (Selleck Chemicals), Erastin (Selleck Chemicals), Ferrostatin-1 (Selleck Chemicals), BHA (Sigma) and Sorafenib (Selleck Chemicals), at indicated concentrations. All molecules were dissolved in DMSO for cell culture experiments. Final concentration of DMSO did not exceed 0.5%.

### Mosquito rearing and sporozoite production

For *P. yoelii* sporozoite production, female 6-8 week old Swiss Webster mice (Harlan, Indianapolis, IN, USA) were injected with blood stage *P. yoelii* (17XNL) parasites to begin the growth cycle. Animal handling was conducted according to the Institutional Animal Care and Use Committee-approved protocols. We used infected mice to feed female *Anopheles stephensi* mosquitoes after gametocyte exflagellation was observed. We isolated salivary gland sporozoites according to the standard procedures at days 14 or 15 post blood meal. For each experiment, salivary glands were isolated in parallel to ensure that sporozoites were extracted from salivary glands under the same conditions.

### Quantification of ROS by Flow cytometry

In total 3.0 × 10^5^ Hepa1-6 cells were seeded in DMEM complete medium in a 24-well plate. Cells were infected with 1.0 × 10^5^ *P. yoelii* sporozoites. The plate was centrifuged for 3 min at 515 × *g* in a hanging-bucket centrifuge to aid in sporozoite invasion. After 90 min, we removed media that contained sporozoites and added fresh media. We allowed LS parasites to develop for 24 or 48 h. One hour prior to the end of the infection, CellROX was added to the cultures according to the manufactures protocol then detached with trypsin and fixed with 4% paraformaldehyde for 10 min. Cells are then blocked with 0.1% Triton X-100 and 2% BSA in PBS for 60 min. Staining steps were performed in PBS supplemented with 0.1% Triton X-100 and 2% BSA. We stained cells using anti-sera to *Plasmodium* CSP conjugated to Pacific Blue at RT in the dark for 60 min and then washed once with PBS+5 mM EDTA. Up to 30,000 live cell events were collected via flow cytometry with an LSRII. Number of infected cells quantified varied from ~60 to 400, depending on infection rate of a given experiment. Data were analyzed using FlowJo software.

### Quantification of lipid peroxidation

In total 1.5 × 10^5^ Hepa1-6 wild type or knockdown cells were seeded in DMEM complete medium in an 8-well chamber slide. Cells were infected with 5 × 10^4^ *P. yoelii* sporozoites. Slides were centrifuged for 3 min at 515 × *g* in a swing-bucket centrifuge to aid in sporozoite invasion and incubated at 37 °C in 5% CO_2_ for 90 min. The infection was washed with PBS and replaced with fresh media containing 50 μM Click-iT linoeamide alkye (ThermoFisher Scientific) and subsequently exposed to Erastin (5 µM in DMSO), Ferrostatin-1 (10 µM DMSO), BHA (5 µM in EtOH) or Nutlin-3 (20 µM in DMSO) for 24 h to develop into exoerythrocytic form (EEF). After incubation, cells were fixed with 3.7% paraformaldehyde, permeabilized with PBS-0.25% Triton- X-100 and blocked with 2% BSA-PBS. *Plasmodium* EEFs were stained with antibodies against *Py*HSP70 and lipid peroxidation was visualized with Alexa fluor 488 azide according to the manufacturer’s protocol. Cells were also stained for nuclei with DAPI and for actin cytoskeleton with Phalloidin and subjected to 3D fluorescent microscopy. Images were acquired with a 100 × 1.4 NA objective (Olympus) on a DeltaVision Elite High Resolution Microscope (GE Healthcare Life Sciences). The sides of each pixel represent 64.5 × 64.5 nm and z-stacks were acquired at 300 nm intervals. Approximately 15–25 slices were acquired per image stack. For deconvolution, the 3D data sets were processed to remove noise and reassign blur by an iterative Classic Maximum Likelihood Estimation widefield algorithm provided by Huygens Professional Software (Scientific Volume Imaging BV, The Netherlands). Imaris software (Bitplane) was used to obtain 3D reconstructions of the fluorescence microscopy image stacks and quantification in *x*-*y*-*z* coordinates. Deconvolved images were processed, thresholded and segmented using Imaris software to render isosurfaces from the fluorescent signals. The surface segmentation function of Imaris was used to identify the cell boundary using the phalloidin signal. The parasite exoerythrocytic forms were segmented using *Py*HSP70 signal. The lipid peroxidation levels of the Hepa1-6 cells and parasite exoerthrocytic forms were measured by quantifying total fluorescent intensity of Alexa-fluor 488 signal normalized to the specific volume of the segmented region of interest.

### Quantification of LS parasites by immunofluorescence in vitro

In total 1.5 × 10^5^ Hepa1-6 cells were seeded in DMEM complete medium in 6-wells of an 8-well Permanox slide that was  treated with a 3.4% Purecol (Advanced BioMatrix) in PBS at 200 µL per well for 1 h, then washed PBS. Cells were infected with 5 × 10^4^ *P. yoelii* sporozoites. Slides were centrifuged for 3 min at 515 × *g* in a hanging-bucket centrifuge to aid in sporozoite invasion. After 90 min, we removed media that contained sporozoites and added fresh media only, or media containing Erastin (10 µM in DMSO), Ferrostatin-1 (10 µM DMSO), Sorafenib (10 µM DMSO), BHA (5 µM in EtOH) or Nutlin-3 (20 µM in DMSO). We allowed LSs to develop for 24 or 48 h, at which time cells were fixed with 10% formalin, blocked, and permeabilized for 1 h in PBS with the addition of 0.1% Triton X-100 and 2% BSA. Staining steps were performed in PBS supplemented with 2% BSA. We stained cells using anti-sera to *Plasmodium* HSP70, which stains the entire parasite cytoplasm, at 4 °C overnight and then washed several times with PBS. Antibodies were visualized with the use of AlexaFluor-488 goat anti-mouse (Invitrogen). We used DAPI to visualize both hepatocyte and parasite nuclei. All LS-infected hepatocytes were counted per well, and each assay was performed in biological triplicate.

### Assessment of Erastin activity in vivo

Twenty female C57Bl/6 mice were treated with either vehicle (10% DMSO in 0.9% Saline) or 30 mg/kg of Erastin for 4 days via oral gavage. On the second day of treatment, all mice were infected through retro-orbital injection with 1000 *P. yoelii* sporozoites. Patency was checked by 10% Giemsa-stained thin blood smear daily beginning on day 3 post-infection, until all mice became patent. Animal handling was conducted according to the Institutional Animal Care and Use Committee-approved protocols.

### NOX1^−/−^ mouse in vivo experiments

NOX1^−/−^ mice (B6.129 × 1-Nox1^tm1Kkr^/J) were obtained from Jackson Laboratory and bred to obtained NOX^−/−^ animals for experiments. C57Bl/6 mice were also obtained from Jackson laboratories and used as controls. Mice were co-housed. For LS burden monitored by qPCR, in each experiment, five C57Bl/6 mice and five NOX1^−/−^ were infected retro-orbital injection with 1.0 × 10^5^ *P. yoelii* sporozoites. A total of 15 C57bl/6 and 15 NOX^−/−^ mice were used across all qPCR experiments. At 44 h, animals were euthanized, and livers were removed. Three independent experiments were performed. To visualize parasites in the liver, C57Bl/6 mice or NOX1^−/−^ mice were infected by retro-orbital injection with 5.0 × 10^5^ *P. yoelii* sporozoites. Livers were fixed with 10% formalin and then transferred to 70% EtOH after 72 h. In total 4 µm sections were stained with Haemotoxylin and Eosin (H&E) using standard methodology. Parasites images were obtained using a phase contrast microscope fitted with a true color camera. Cross-sectional areas were measured using Nikon Image Software-Elements Advanced Research Analysis Software version 4.5. Liver slice areas were measured using ImageJ software. Experiment was performed two independent times.

### Primary Hepatocyte isolation and culture

Hepatocytes were isolated from 8-week-old C57Bl/6 or C57Bl/6 NOX1^−/−^ mice according to previously described methods [17]. Briefly, mice were anesthetized with isoflurane and the portal vein was exposed and cannulated with a 26G catheter. The liver was perfused and digested with collagenase type IV (Sigma). Hepatocytes were then purified via Percoll centrifugation and seeded at a density of 1.5 × 10^5^ hepatocytes per well onto 48-well plates coated with 0.17 mg/ml rat tail Collagen-1 (BD Biosciences). Hepatocytes were cultured in media containing DMEM with high glucose (4.5 g/L), 10% (v/v) fetal bovine serum (Biowest), 0.04 µg/ml dexamethasone, 7 ng/ml glucagon, 1% ITS+culture supplement (Corning), 1.5% 1 M HEPES, and 1% penicillin-streptomycin. The next day, hepatocytes were infected. Replicate experiments were performed with both male and female mice.

### Quantification of liver burden by real-time RT-PCR

Total RNA was extracted using TRIzol reagent (Invitrogen). cDNA synthesis was performed using the Prime Script RT reagent kit with gDNA Eraser quantitative reverse transcription-PCR (qRT-PCR) kit according to the manufacturer’s instructions (Takara-Clonetech). All PCR amplification cycles were performed at 95 °C for 30 s for DNA denaturation and 60 °C for 4 min for primer annealing and DNA strand extension. Parasite 18S was amplified using primers with sequences 5′-GGGGATTGGTTTTGACGTTTTTGCG-3′ and 5′-AAGCATTAAATAAAGCGAATACATCCTTAT-3′. Mouse glyceraldehyde-3-phosphate dehydrogenase (GAPDH) was amplified using sequences 5′-CCTCAACTACATGGTTTACAT-3′ and 5′-GCTCCTGGA AGATGGTGATG-3′. For quantitative PCR (qPCR), mouse SLC7a11 was amplified using the sequences 5′ – CTTTGTTGCCCTCTCCTGCTTC – 3′ and 5′ – CAGAGGAGTGTGCTTGTGGACA – 3′. Knockdown levels of SLC7all were determined by qRT-PCR. Data were normalized to GAPDH and a scramble control. Fold change was determined using the 2^−ΔΔct^ method.

### Quantification of p53 by Western Blotting

In total 1 × 10^6^ Hepa1-6 cells were plated per well of a 6-well plate in DMEM complete media and treated with complete media only, 20 µM Nutlin-3 or 10 µM Erastin for 24 h. Cells were lysed in SDS lysis buffer (2% SDS, 50 mM Tris-HCl, 5% glycerol, 5 mM EDTA, 1 mM NaF, 10 mM β-glycerophosphate, 1 mM PMSF, 1 mM activated Na_3_VO_4_, 1 mM DTT, 1% phosphatase inhibitor cocktail 2 (Sigma-Aldrich), 1% PhosSTOP Phosphatase Inhibitor Cocktail Tablet (Roche)), filtered overnight at 3000 rpm through AcroPrep Advance Filter Plates (Pall Corporation), and stored at −80 °C. Western blots were performed according to manufacturer instruction with the iBlot Dry Transfer System (Life Technologies, Carlsbad, CA, USA) using an antibody to p53 (Clone 1C12; Cell Signaling Technology) and then normalized to signal from an anti-β-actin (Cell Signaling Technology). Signals from immunoblots were detected using either an Alexa 680-conjugated anti-rabbit antibody or an Alexa 800-conjugated anti-mouse antibody (LI-COR Biosciences). Membranes were visualized using an Odyssey infrared imaging system (LI-COR Biosciences).

### CRISPR design and generation

Single guide RNAs (sgRNAs) for SLC7a11 were designed using E-CRISP [18]. Two sgRNAs with single target specificity and high efficiency that fell within the first translated exon were selected. sgRNAs were ligated into the digested lentiCRISPRv2 backbone using the protocol from Shalem et al. [19]. Briefly, oligos for SLC7a11 that spanned the sgRNA sequence were annealed and the 5′ ends phosphorylated as shown in the table below. Annealed oligos were ligated into BsmBI (ThermoFisher) digested lentiCRISPRv2 and transformed into Stbl3 (ThermoFisher) bacteria. Plasmids were isolated using the Nucleobond Xtra Maxi Kit (Takara) and sequenced using the U6 primer (5′-GACTATCATATGCTTACCGT-3′; Genewiz).

**Table:** 

sgRNA	CRISPR Sequence (5′→ 3′)	TIDE Primer Sequence (forward): (5′→ 3′)	TIDE Primer Sequence (reverse): (5′→ 3′)	Knockdown efficiency (TIDE)	Knockdown efficiency (Western Blot)
Nontargeting sgRNA	CTGTCTTCAACGTCTGGCCG				
SLC7a11 sgRNA#1	GGACCAAGAGCCACCTGGGC	TGTAGAGCCAGTCGGTGATAGC	GGGTAGTGCACATACCTGAACAAC	52.6%	46%
SLC7a11 sgRNA#2	GATGTAGCGTCCAAATGCCA	CCTCAAACCTTGTGTTCCTGTCTG	CTGGATTGCTATCTTCACAGGCC	49.5%	59%

### Lentivirus production

MISSION shRNA plasmids were obtained from Sigma Aldrich (St. Louis, MO) and lentiCRISPR vectors were generated in house, as described above. Detailed information on shRNA constructs can be found in Table S1. Nonreplicating lentiviral stocks were produced by transfection of HEK293-FT cells. TC-treated petri dishes (10 cm) were coated with 0.01 mg/mL poly-L-lysine for ≥30 min at 37 °C and rinsed with diH_2_O twice. In total 4 × 10^6^ HEK293-FT cells were plated on poly-L-lysine coated dishes to achieve 70–80% confluency at time of transfection. Approximately 24 h after plating, transfection mixtures were prepared by mixing 20 µl Polyethylenimine MAX (Polysciences Inc,Warrington, PA) prepared at 1 mg/ml, together with 4.75 µg of transgene (shRNA construct, non-targeting shRNA control, or lentiCRISPR construct), 1.5 µg viral envelope plasmid (pCMV-VSV-G), and 3.75 µg viral packaging plasmid (psPax2). After incubating for 10 min at room temp in DMEM, transfection complexes were added drop-wise to cells. The following morning, cells were washed 1× to remove transfection mixtures and were fed with 10 ml fresh media. Lentivirus-containing supernatant was harvested 36 h later, passed through 0.45 µm syringe filters, and either used immediately for transduction or stored at −80 °C.

### Lentiviral transduction

**Fig. 1:**
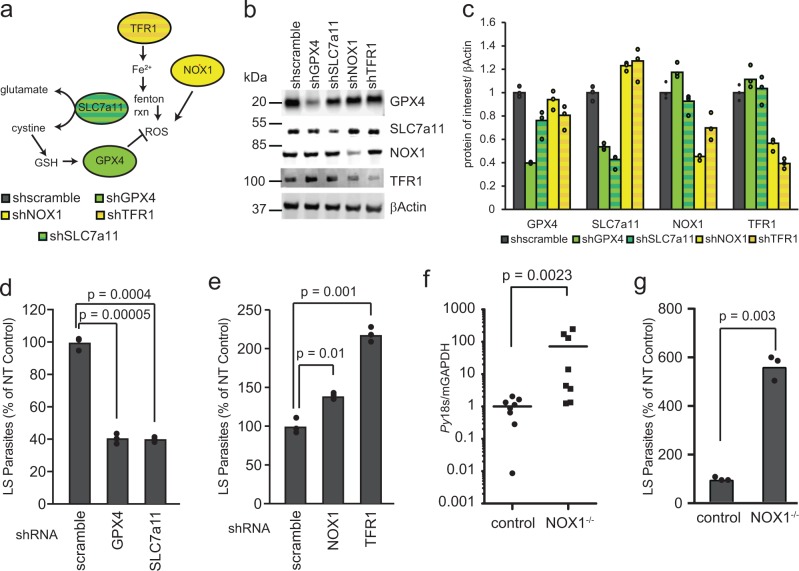
SLC7a11 signaling is a potent regulator of LS *Plasmodium* infection. **a** Schematic of SLC7a11-driven signaling as reported in the literature. **b**, **c** Hepa1-6 cells were transduced with 2–4 lentivirus expressing shRNAs against each gene of interest. Cellular lysates were isolated after lentivirus transduction and selection with puromycin and western immunoblots were performed against the indicated targets. Signal was normalized to βActin. **d**, **e** Hepa1-6 cells were transduced with lentivirus expressing shRNAs against **d** GPX4, SLC7a11 or **e** NOX1 and TFR1 as well as a non-targeting shRNA control (scramble). 1.5 × 10^5^ cells were infected with 5 × 10^4^ *P. yoelii* sporozoites and quantified by microscopy. **f** WT or NOX1^(−/−)^ C57Bl/6 mice were infected via retro-orbital injection with 10^5^ *P. yoelii* sporozoites. Livers were isolated 42 h post-infection and LS burden was quantified by qRT-PCR. **g** WT or NOX1^(−/−)^ C57Bl/6 mice were perfused and primary hepatocytes from each strain of mouse were isolated. 1.5 × 10^5^ Primary hepatocytes were infected with 10^5^ *P. yoelii* sporozoites. Infections were quantified by microscopy

To induce knockdown of candidate host kinases, Hepa1-6 cells were transduced with lentiviral supernatants in 6-well plates at 1 × 10^6^/well. At time of plating, cells were transduced with 1 ml of supernatant in the presence of 0.5 µg/mL polybrene. In order to select for cells with stable integration of shRNA transgenes, media was replaced with selection media (complete media with the addition of 2 µg/mL puromycin) 24 h post transduction, and cells were selected for 3–5 days prior to experiments. For lentiCRISPR perturbation, Hepa1-6 cells were seeded into 6-well plates at a density of ~50% at the time of transduction. Supernatants were added to respective wells in 2 mL media containing 10 µg/mL polybrene (Santa Cruz Biotechnology). After 24 h, wells were washed and replaced with fresh media containing 1 µg/mL puromycin. After 3 days selection, media was replaced, and selected colonies were expanded. Knockdown for shRNA constructs was validated via western blot (Fig. 1a). Antibodies were used at the following dilutions: TFR1 at 1:1000 (Abcam ab1086), GPX4 at 1:2500 (Abcam ab125066), SLC7a11 at 1:1000 (Abcam ab37185), and β-Actin at 1:2000 (Cell Signaling #4970 and #3700). Disruptions for CRISPR constructs were validated via TIDE sequencing [20]. Genomic DNA was isolated using AllPrep DNA/RNA Mini Kit (Qiagen). Region surrounding target was amplified with Herc II polymerase (Agilent) using primers in the table below. PCR products were purified via gel extraction. TIDE sequencing analysis was performed using the Netherlands Cancer Institute analysis tool (https://tide-calculator.nki.nl/). The primers used for each CRISPR construct are shown below. Sequences for shRNAs are included in Table S1.

### *Plasmodium* blood stage assay

GFP-Luciferase expressing *Plasmodium falciparum* blood stage parasites [21] were cultured asexually. Assays were performed on synchronous (>90%) ring stage parasites at 2% parasitemia and 5% hematocrit. In triplicate, Erastin was administered to parasites at different titrations with vehicle as a non-treatment control (1% DMSO in complete media), and Chloroquine administered at 2.5 µM as a positive control. At 44 h post treatment, 30 µL of the assay culture was transferred to 96-well white flat-bottom opaque tissue culture plate (Beckton Dickinson, Franklin Lakes, NJ). Using a Berthold LB960 XS3 microplate (Berthold Technologies, Wildbad, Germany) an equal volume of Bright-Glo luciferase reagent (Promega, Madison, WI) was added to parasites and resultant luminescence was measured. Experimental data is representative of three biological replicates.

## Results

To probe the capacity of the SLC7a11 pathway to regulate *Plasmodium* liver stage infection, we generated knockdown hepatoma cell lines for several components of the pathway. Specifically, Hepa1-6 cells were transduced with lentiviruses expressing shRNAs against SLC7a11, and glutathione peroxidase 4 (GPX4), positive regulators of the pathway, or NADPH oxidase 1 (NOX1) and transferrin receptor protein 1 (TFR1), negative regulators of the pathway [15, 22] (Fig. 1a–c). A significant decrease in the number of LS parasites was observed after 24 h in SLC7a11 and GPX4 knockdown cells (Fig. 1d). In contrast, infection of TFR1 or NOX1 knockdown cell lines resulted in significantly increased numbers of LS parasites (Fig. 1e). To examine whether this pathway alters LS burden in vivo, and to evaluate the impact of complete NOX1 knockout, we compared infection in WT and NOX1^−/−^ C57Bl/6 mice. NOX1^−/−^ mice exhibited a dramatic and statistically significant increase in LS burden when infection was assessed by PCR of *P. yoelii* 18s rRNA (Fig. 1f). To determine if the impact of NOX1^−/−^ on LS parasites was hepatocyte-specific, we isolated primary hepatocytes from WT or NOX1^−/−^ mice and infected primary hepatocyte cultures with *P. yoelii* sporozoites, and let infection proceed for 24 h. Again, a significant increase in the number of LS parasites was observed in NOX1^−/−^ hepatocytes (Fig. 1g). Taken together, reducing levels of SLC7a11 or GPX4 reduces LS infection, whereas reducing NOX1 or TFR1 levels increases LS infection.

**Fig. 2:**
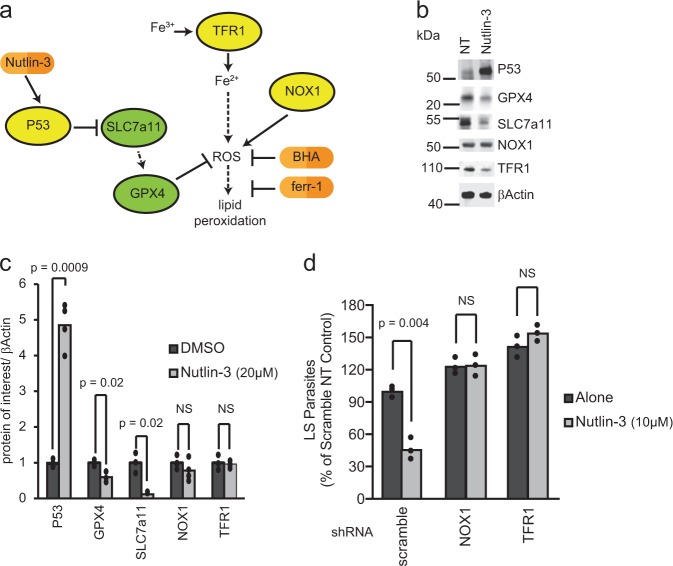
SLC7a11 pathway is responsible for P53-mediated elimination of LS-infected hepatocytes. **a** Schematic of signaling induced by P53. **b**, **c** 1.5 × 10^5^ Hepa1-6 cells were treated with 10 µM Nutlin-3. Cellular lysates were isolated 24 h after treatment and immunoblots against the indicated targets were performed. Signal was quantified using Image Studio software and normalized to βActin. **d** 1.5 × 10^5^ Hepa1-6 cells were transduced with lentivirus expressing shRNAs against a scramble control, NOX1 or TFR1 and infected with 5 × 10^4^ *P. yoelii* sporozoites. After 90 min post-infection, cells were treated with 10 µM Nutlin-3 or a DMSO control. Parasites were visualized 24 h post-infection by Hsp70 staining and quantified by microscopy. Each bar represents the mean of replicates. *P*-values were obtained using a Student’s *t*-test

The ability of P53 to act as a potent tumor suppressor, independent of its roles in initiation of apoptosis, cell cycle arrest, and senescence, is thought to depend on its capacity to block SLC7a11 and, subsequently, generate ROS and lipid peroxides (Fig. 2a) [23]. We have previously demonstrated that elevated levels of P53 reduce LS burden, independent of the role of P53 in apoptosis, and likely independent of its role in promoting cell cycle arrest [12, 13, 24, 25]. The small molecule Nutlin-3 binds the E3 ubiquitin ligase MDM-2, which under normal conditions acts to degrade P53, thereby increasing P53 protein levels [26]. We have previously demonstrated that P53 is required for the reduction in Plasmodium infection after Nutlin-3 treatment [12, 13]. To evaluate how elevated levels of P53 impacts the SLC7a11-GPX4 pathway, Hepa1-6 cells were treated with 10 µM Nutlin-3 for 24 h and levels of SLC7a11, GPX4, NOX1 and TFR1 were evaluated by Western Blot. Treatment with Nutlin-3 reduced SLC7a11 and GPX4 protein levels (Fig. 2b, c). We reasoned that if P53 acted to eliminate LS parasites through the SLC7a11 pathway, we could modulate its effect with the knockdown of NOX1 or TFR1. Hepa1-6 cells transduced with a control shRNA or shRNAs directed against TFR1 or NOX1 and then were infected with *P. yoelii* sporozoites for 90 min. After 90 min, Nutlin-3 was added for 24 h. We observed a complete loss of susceptibility of LS-infected cells to Nutlin-3 treatment when treatment occurred in the context of TFR1 or NOX1 knockdown (Fig. 2d), suggesting that the ability of Nutlin-3 to eliminate LS parasites is dependent on the SLC7a11 pathway.

**Fig. 3:**
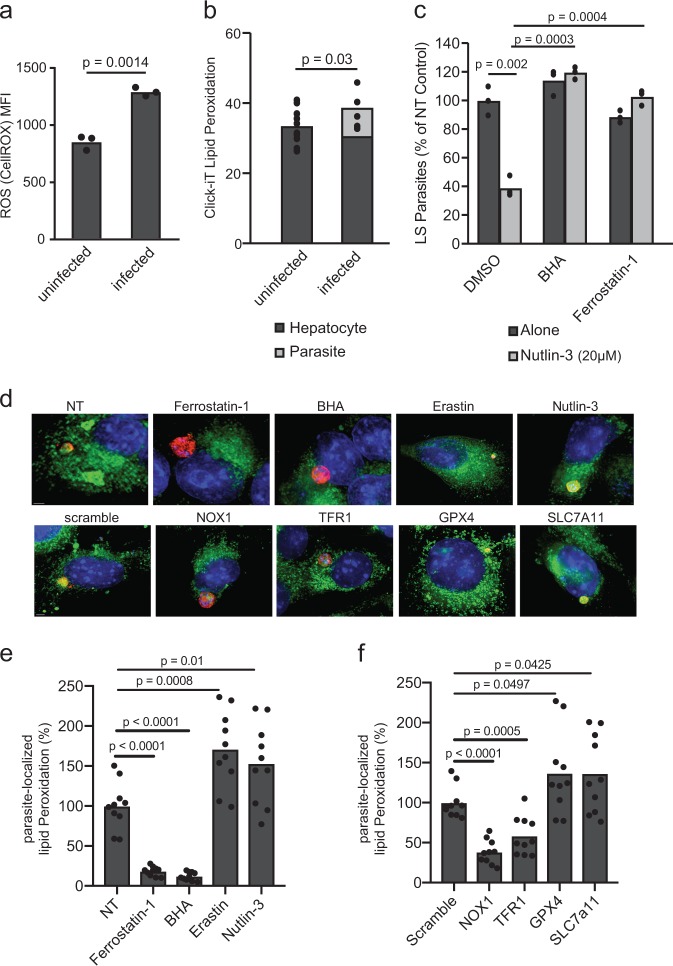
Reactive oxygen species (ROS) and lipid peroxidation are key mediators of SLC7a11 pathway control of LS infection. **a** Hepa1-6 cells were infected with 1.5 × 10^5^ *P. yoelii* sporozoites and then evaluated for ROS with a CellROX dye 24 h after infection via flow cytometry. Mean fluorescent intensity (MFI) of signal obtained is presented. P-value was obtained using a Student’s *t*-test. **b** Hepa1-6 cells were infected with 1.5 × 10^5^ *P. yoelii* sporozoites and then evaluated for lipid peroxidation with Click-iT Lipid dye 24 h post-infection by fluorescence microscopy. Data are shown as fluorescence intensity normalized to area in infected and uninfected cells. Fluorescent signal was identified as localizing to the hepatocyte or to the parasite. Each dot represents a single cell. **c** 1.5 × 10^5^ Hepa1-6 cells were infected with 5 × 10^4^ *P. yoelii* sporozoites. 90 min post-infection, cells treated with a DMSO control, 20 µM Nutlin-3, 5 µM BHA, or 300 nM ferrostatin-1 as indicated. Parasites were visualized by *Py* HSP70 staining 24 h post-infection and quantified by microscopy. In all panels, points represent individual analytical replicates. **d** Hepa1-6 cells, in the context of knockdown or drug treatment as indicated, were infected with 1.5 × 10^5^ *P. yoelii* sporozoites and then evaluated for lipid peroxidation with Click-iT Lipid dye 24 h post-infection by fluorescence microscopy. Click-iT Lipid dye and drug treatments were added 90 min post-infection. Drug treatments include Erastin (5 µM), Ferrostatin-1 (10 µM), BHA (5 µM), and Nutlin-3 (20 µM). Representative merged images of hepatocytes 24 h after infection with *P. yoelii* are shown. DAPI is shown in blue, *Py* HSP70 in red, and lipid peroxides in green. The scale bar is 2 µm. **e** Quantification of parasite-localized lipid peroxidation, normalized to area, 24 h post-infection, in the context of drug treatment. Data were normalized to the NT mean. Each point represents a single infected cell. **f** Quantification of parasite-localized lipid peroxidation, normalized to area, 24 h post-infection, in the context of each knockdown. Data were normalized to the scramble. Each point represents a single infected cell. P-values were obtained using a Student’s *t*-test

It has been previously demonstrated that P53 inhibition of SLC7a11 induces ROS production, and subsequently, lipid peroxidation [23]. We monitored ROS in *P. yoelii*-infected and uninfected Hepa1-6 cells following 24 h infection. *P. yoelii*-infected cells exhibited elevated ROS levels (Fig. 3a), suggesting that either a parasite-intrinsic response or a host-defense promotes ROS production in the context of infection. We next asked if lipid peroxides were generated during infection. To do this, we again infected Hepa1-6 cells with *P. yoelii* sporozoites and evaluated lipid peroxide levels using a Click-iT lipid peroxide probe. Interestingly, we observed high levels of lipid peroxidation within the parasite (Fig. 3b, Fig. S1), yet no difference in lipid peroxide levels between infected and uninfected cells excluding the parasite-localized signal (Fig. 3b). To determine if the observed anti-parasitic activity of Nutlin-3 is ROS and/or lipid peroxidation dependent, Hepa1-6 cells were infected with *P. yoelii* sporozoites, then treated with Nutlin-3 in the presence or absence of a ROS scavenger, butylated hydroxyanisole (BHA) or an inhibitor of lipid peroxidation, ferrostatin-1. Both BHA and ferrostatin-1 reversed the anti-parasitic activity of Nutlin-3 (Fig. 3c), demonstrating that the activity of Nultin-3 requires ROS and lipid peroxidation. Similarly, the addition of BHA or ferrostatin-1 partially reversed the effect of SLC7a11 or GPX4 knockdown (Fig. S2).

Next, we evaluated parasite-localized lipid peroxidation in response to genetic and pharmacological alterations in the SLC7a11 pathway (Fig. 3d). In response to knockdown of SLC7a11 or GPX4, or treatment with Erastin or Nutlin-3, we observe an increase in levels of parasite-localized lipid peroxides (Fig. 3e, f). In response to BHA or Ferrostatin-1 treatment, or in response to NOX1 or TFR1 knockdown, we observe a decrease in parasite-localized lipid peroxides (Fig. 3e, f). Taken together, these results suggest that the role of P53 in curtailing malaria liver stage infection depends on ROS production and lipid peroxidation, and further establishes the non-canonical roles of P53 as critical for liver stage infection.

**Fig. 4:**
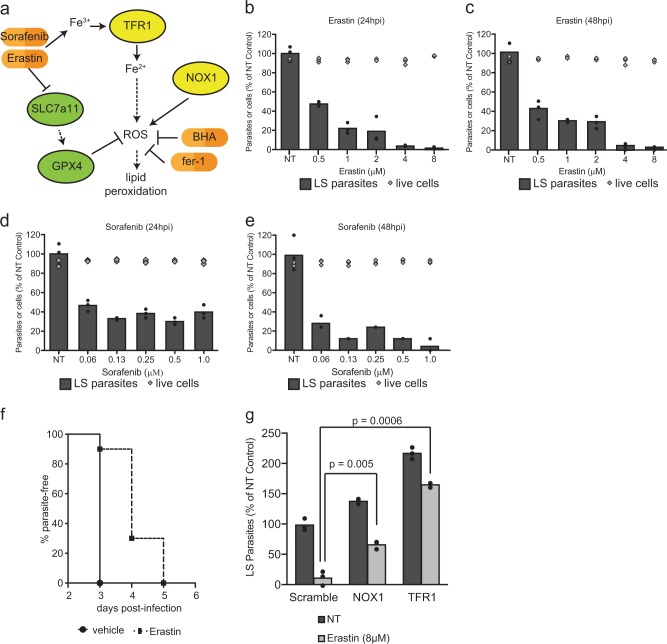
Induction of ferroptosis-like signaling with small molecules eliminates *Plasmodium* LS parasites in vitro and in vivo. **a** Signaling downstream of Erastin treatment as reported in the literature. **b**, **c** Hepa1-6 cells were infected with 5 × 10^4^ *P. yoelii* sporozoites and treated with Erastin at indicated concentrations 90 min after infection. After **b** 24 h or **c** 48 h, LS parasites were visualized by *Py* HSP70 staining and quantified by fluorescent microscopy. Cell death in uninfected cells was quantified by Trypan Blue staining. **d**, **e** Hepa1-6 cells were infected with *P. yoelii* and treated with Sorafenib at indicated concentrations. After 24 h (**d**) or 48 h (**e**), LS parasites were visualized by *Py*HSP70 staining and quantified by fluorescent microscopy. Cell death in uninfected cells was evaluated by Trypan Blue staining. **f** 10 C57Bl/6 mice were treated with 30 mg/kg Erastin or vehicle control for 4 days. On the second day of treatment, mice were challenged with 1000 *P. yoelii* sporozoites by retro-orbital injection. Beginning on day 3 post-infection, blood was evaluated by giemsa-stained thin smear for the presence of blood stage parasites. Each bar represents the mean of replicates. **g** 1.5 × 10^5^ Hepa1-6 cells transduced with lentivirus expressing shRNAs against a scramble control, NOX1 or TFR1 and infected with 5 × 10^4^ *P. yoelii* sporozoites. After 90 min post-infection, cells were treated with 8 µM Erastin or a DMSO control. Parasites were visualized 24 h post-infection by Hsp70 staining and quantified by microscopy. Each bar represents the mean of replicates. *P*-values were obtained using a Student’s *t*-test. In **a**–**e** and **g**, points represent individual technical replicates and are representative of three independent experiments

The P53-dependent production of ROS has been associated with ferroptosis, a ROS, lipid peroxide, and iron-dependent cell death. Ferroptosis was originally described as a form of cell death mediated by Erastin, a small molecule that has been shown to inhibit SLC7a11 [15]. More recently, ferroptosis has been demonstrated to occur in other contexts and to lead to cell death through peroxidation of host lipids [16, 27–31]. This finding has led to the development of several small molecules inhibitors that target the SLC7a11 pathway. In order to determine if LS infection was susceptible to the pharmacological inhibition of SLC7a11 (Fig. 4a), Hepa1-6 cells were infected with *P. yoelii* sporozoites, then treated with Erastin begining at 1.5 h post-infection. A substantial, dose-dependent reduction in the number of LS parasites at 24 h and 48 h was measured following infection, but there was no increase in cell death of uninfected cells across the same range of Erastin concentrations (Fig. 4b, c).

While Erastin treatment is a potent inhibitor of SLC7a11, it also has other targets, which might also be capable of reducing parasite burden [32]. Thus, we asked if other inhibitors of this pathway also reduce parasite burden. Infected hepatocytes were treated with Sorafenib, another SLC7a11 inhibitor [33–35], which resulted in a similar decrease in parasite burden (Fig. 4d, e). Neither Erastin nor Sorafenib impacted the growth of *Plasmodium*-infected erythrocytes (Fig. S3) [36], suggesting these inhibitors act by targeting a host molecule rather than directly targeting a parasite pathway. Moreover, a third, structurally-unrelated inhibitor of the pathway (specifically, a GPX4 inhibitor) RSL3, also reduced the number of liver stage parasites without causing cell death in uninfected Hepa1-6 cells (Fig. S4). Finally, the effect of Erastin was completely ablated in cells that were transduced with sgRNAs against SLC7a11 (Fig. S5). Thus, Erastin requires SLC7a11 for its activity against LS parasites.

We next asked if LS-infected hepatocytes in vivo were sensitive to Erastin. C57bl/6 mice were treated with 30 mg/kg Erastin for 4 days, then challenged with *P. yoelii* sporozoites on the second day of Erastin treatment. We reasoned that any decrease in LS infection would result in a delay in the onset of blood stage infection. Blood stage patency was monitored by thin smear in mice, and results showed a 1-2-day delay in the onset of blood stage infection (Fig. 4f).

Finally, we explored the mechanism of action of Erastin on LS infection. Treatment of infected cells with Erastin increased the intensity of lipid peroxidation staining that localized to the parasite (Fig. 3d, e). Finally, in the context of NOX1 or TFR1 knockdown, both of which decreased parasite-localized lipid peroxides (Fig. 3d, f), we observed a substantial decrease in the efficacy of Erastin on LS infection (Fig. 4g). Thus, both pharmacological and genetic perturbations to signal transduction cascades that are known to generate ROS and to promote lipid peroxidation, result in parasite-localized lipid peroxides and reduce LS parasite levels.

## Discussion

The mechanisms by which hepatocyte signaling regulates liver stage parasite infection remains an area of nascent exploration. Here, we show that the hepatocyte SLC7a11-GPX4 signaling pathway modulates *Plasmodium* infection through the generation of ROS and lipid peroxidation. The effect of knockdown of host factors, not present in the parasite, on LS infection and parasite-localized lipid peroxidation suggests that ROS production and lipid peroxidation are, at least partially, driven by host cell signaling. It is also possible that the *Plasmodium* parasite itself is susceptible to ferroptosis-like cell death, as has been described in trypanosomes [37]. Whether the lipids that undergo peroxidation are of host and/or parasite origin, and how this leads to parasite clearance, are important topics for future investigation.

P53 has been previously demonstrated to block SLC7a11 [23]. Moreover, survival of multiple pathogens has been linked to decreased levels of P53 within the infected cell (reviewed in [38]). We and others have previously demonstrated that the role of P53 in curtailing malaria infection is not dependent on its conventional roles in apoptosis [13]. Here, we demonstrate that the ability of P53 to control LS infection is instead dependent on ROS and the generation of lipid peroxides. As such, our work further establishes non-canonical activities of P53 as critical for the control of aberrant signaling states. Future work will likely establish whether the reach of this signal transduction cascade extends beyond LS malaria infection and operates to control other pathogens.

The expansion and compression of pathogen numbers are a hallmark of infectious lifecycles, including malaria. For many intracellular pathogens, attrition occurs as the result of host cell death mechanisms like apoptosis, necrosis, or pyroptosis. In addition to serving as innate defenses, these cell death mechanisms also inform subsequent adaptive responses (reviewed in [39]). It will be interesting to ask what role LS parasites that are eliminated by the generation of lipid peroxides play in shaping a subsequent immune response to *Plasmodium* liver infection. It was recently described in an elegant set of experiments that transcriptomic responses, along with cell phenotypic responses during cell death, could inform adaptive responses to infection [40]. This finding is particularly relevant as evidence mounts that the heterogeneity across cells might be much greater than originally appreciated [41–43]. Indeed, the innate and adaptive systems that control infection may engage a broader range of molecular processes, including the generation of lipid peroxides, than we have traditionally incorporated into our understanding of immunity.

## Supplementary information

Supplemental Table 1

Supplemental figure legends

Supplemental Figure 1

Supplemental Figure 2

Supplemental Figure 3

Supplemental Figure 4

Supplemental Figure 5
