# Supplementary material for: Liver stage malaria infection is controlled by host regulators of lipid peroxidation
Source: Cell Death Differ. 2019 May 7;27(1):44–54. doi: 10.1038/s41418-019-0338-1 (PMC7206113; doi:10.1038/s41418-019-0338-1)
Supplement: Supplementary file 1 — Supplemental Table 1 [file 41418_2019_338_MOESM1_ESM.docx]

**Supplemental Table 1. Sequences of shRNA constructs used in this study**

| PLASMID | SOURCE | IDENTIFIER |
| --- | --- | --- |
| psPAX2 | Gift from Didier Trono (Addgene plasmid # 12260) | Addgene plasmid #12260 |
| pCMV-VSV-G | Gift from Bob Weinberg (Steward et al., 2003) | Addgene plasmid #8454 |
| control shRNA - CCGGCAACAAGATGAAGAGCACCAACTCGAGTTGGTGCTCTTCATCTTGTTGTTTTTG | Sigma | SHC002 |
| SLC7A11 – shRNA - CCGGCCCTGCATATTATCTCTTCATCTCGAGATGAAGAGATAATATGCAGGGTTTTTG | Sigma | TRCN0000079425 |
| SLC7A11 shRNA - CCGGCCGGAAATCCTCTCTATGATTCTCGAGAATCATAGAGAGGATTTCCGGTTTTTG | Sigma | TRCN0000079427 |
| SLC7A11 shRNA - CCGGGCCCTGTCCTATGCAGAATTACTCGAGTAATTCTGCATAGGACAGGGCTTTTTG | Sigma | TRCN0000079426 |
| SLC7A11 shRNA - GTACCGGCTCTTCATCCCGGCACTATTTCTCGAGAAATAGTGCCGGGATGAAGAGTTTTTTG | Sigma | TRCN0000381330 |
| SLC7A11 shRNA -  CCGGTGGGTGGAACTGCTCGTAATACTCGAGTATTACGAGCAGTTCCACCCATTTTTG | Sigma | TRCN0000311401 |
| GPX4 shRNA - CCGGCCGGCTACAACGTCAAGTTTGCTCGAGCAAACTTGACGTTGTAGCCGGTTTTTG | Sigma | TRCN0000235004 |
| GPX4 shRNA - CCGGGCCAGGAAGTAATCAAGAAATCTCGAGATTTCTTGATTACTTCCTGGCTTTTTG | Sigma | TRCN0000235003 |
| GPX4 shRNA - CCGGGTCGATCTGCATGCCCGATATCTCGAGATATCGGGCATGCAGATCGACTTTTTG | Sigma | TRCN0000235002 |
| GPX4 shRNA - CCGGACAGCAAGATCTGTGTAAATGCTCGAGCATTTACACAGATCTTGCTGTTTTTTG | Sigma | TRCN0000235005 |
| GPX4 shRNA - CCGGATGCCATCAAATGGAACTTTACTCGAGTAAAGTTCCATTTGATGGCATTTTTTG | Sigma | TRCN0000235006 |
| NOX1 shRNA - CCGGGATAGCAACATTGCTGGTCATCTCGAGATGACCAGCAATGTTGCTATCTTTTTG | Sigma | TRCN0000222686 |
| NOX1 shRNA - CCGGCGTGATTACCAAGGTTGTCATCTCGAGATGACAACCTTGGTAATCACGTTTTTG | Sigma | TRCN0000076600 |
| NOX1 shRNA - CCGGGAAAGAAGATTCTTGGCTAAACTCGAGTTTAGCCAAGAATCTTCTTTCTTTTTG | Sigma | TRCN0000076601 |
| NOX1 shRNA - CCGGCTTGAAATCTATCTGGTACAACTCGAGTTGTACCAGATAGATTTCAAGTTTTTG | Sigma | TRCN0000222685 |
| TFRC shRNA - CCGGCGTTGAATTGAACCTGGACTACTCGAGTAGTCCAGGTTCAATTCAACGTTTTTG | Sigma | TRCN0000032256 |
| TFRC shRNA - CCGGGTGATATCTTCTCAGATTATCCTCGAGGATAATCTGAGAAGATATCACTTTTTG | Sigma | TRCN0000375695 |

**Table. S1. Sequences of shRNA constructs used in this study.** Table of shRNA constructs used to make lentiviral knockdowns for GPX4, SLC7a11, NOX1, and TFR1.
