# Supplementary material for: Liver stage malaria infection is controlled by host regulators of lipid peroxidation
Source: Cell Death Differ. 2019 May 7;27(1):44–54. doi: 10.1038/s41418-019-0338-1 (PMC7206113; doi:10.1038/s41418-019-0338-1)
Supplement: Supplementary file 2 — Supplemental figure legends [file 41418_2019_338_MOESM2_ESM.docx]

**Supplemental figure legends**

**Fig S1. Lipid peroxidation localizes to the parasite during LS infection.** Representative images of hepatocytes 24 hours after infection with *P. yoelii* in the context of (a) knockdown or (b) drug treatment as indicated. Data are representative of 2 and 3 biological replicates, respectively. DAPI is shown in blue, *Py* HSP70 in red, and lipid peroxides in green. The scale bar is 2 µm.

**Fig S2. Effects of GPX4 and SLC7a11 knockdown on LS infection are reversed by inhibition of ROS and lipid peroxidation.** 1.5×10^5^ Hepa1-6 cells were transduced with lentivirus expressing shRNAs against a scramble control, GPX4 or SLC7a11 and infected with 5×10^4^ *P. yoelii* sporozoites. 90 minutes post-infection, cells were treated with 5 µM BHA, 300nM Ferrostatin-1, or a control. Parasites were visualized 24 h post-infection by Hsp70 staining and quantified by microscopy.

**Fig. S3. Treatment with Erastin does not impact *Plasmodium* asexual blood stage infection.** Luciferase-expressing *Plasmodium falciparum* blood-stage parasites were cultured asexually and synchronized to 2% ring stage parasitemia. Cultures were treated with Erastin at indicated concentrations. Luciferase activity was measured after 48 h. Points represent individual analytical replicates.

**Fig. S4. RSL3 treatment eliminates LS parasites but does not induce cell death in uninfected cells.** 1.5×10^5^ Hepa1-6 cells were infected with 5.0×10^4^ *P. yoelii* sporozoites. 90 minutes after infection, cultures were treated with either a DMSO as a control or 330 nM RSL-3 and 24 h after treatment LS infection was evaluated with visualized *Py* HSP70 staining and quantified by microscopy. Cell death in uninfected cells was evaluated by Trypan Blue staining.

**Fig. S5. CRISPR/Cas9 mediated knockout of SLC7a11 reduces the number of liver stage parasites.** Hepa1-6 cells were transduced with control or SLC7a11 sgRNA constructs. (a) 1.5×10^5^ Hepa1-6 cells of each line were infected with 5.0×10^4^ *P. yoelii* sporozoites. LS infection was evaluated with visualized *Py* HSP70 staining and quantified by microscopy. (b) 1.5×10^5^ Hepa1-6 cells of each line were infected with 5.0×10^4^ *P. yoelii* sporozoites. 90 minutes after infection, cultures were treated with either a DMSO as a control or 8 µM of Erastin and 24 h after treatment LS infection was evaluated with visualized *Py* HSP70 staining and quantified by microscopy.
