# Supplementary figures and images for: Liver stage malaria infection is controlled by host regulators of lipid peroxidation
Source: Cell Death Differ. 2019 May 7;27(1):44–54. doi: 10.1038/s41418-019-0338-1 (PMC7206113; doi:10.1038/s41418-019-0338-1)

Figure S1

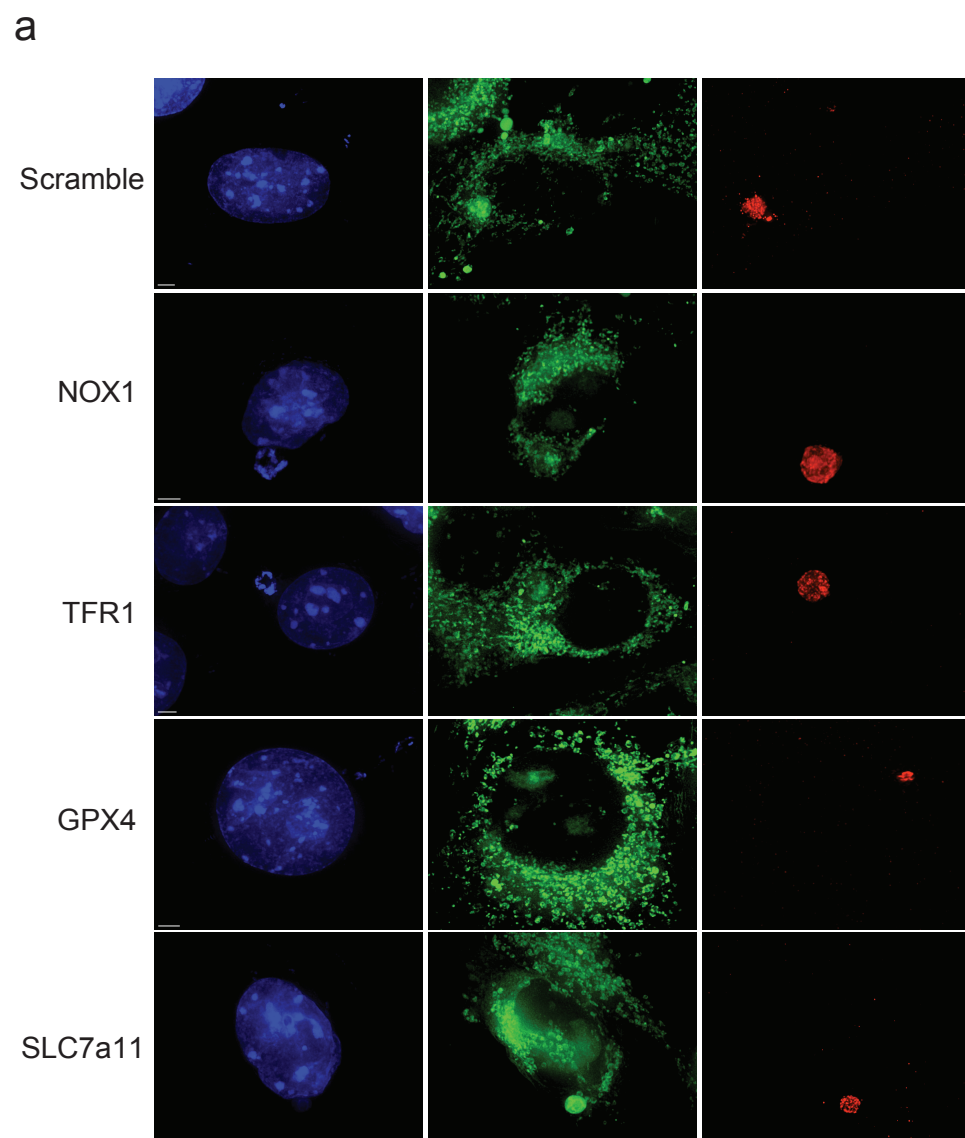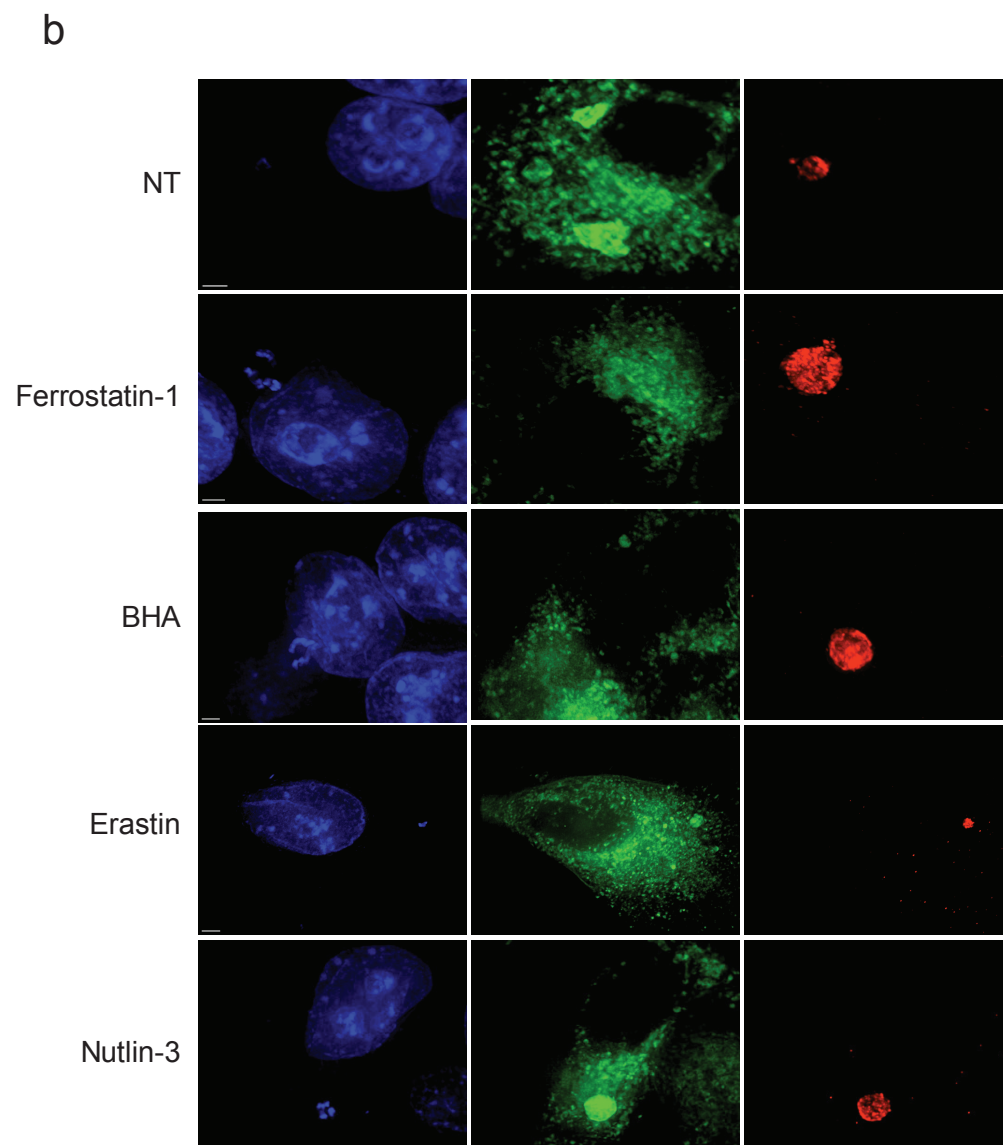

Supplement: Supplementary file 3 — Supplemental Figure 1 [file 41418_2019_338_MOESM3_ESM.pdf]

Figure S2

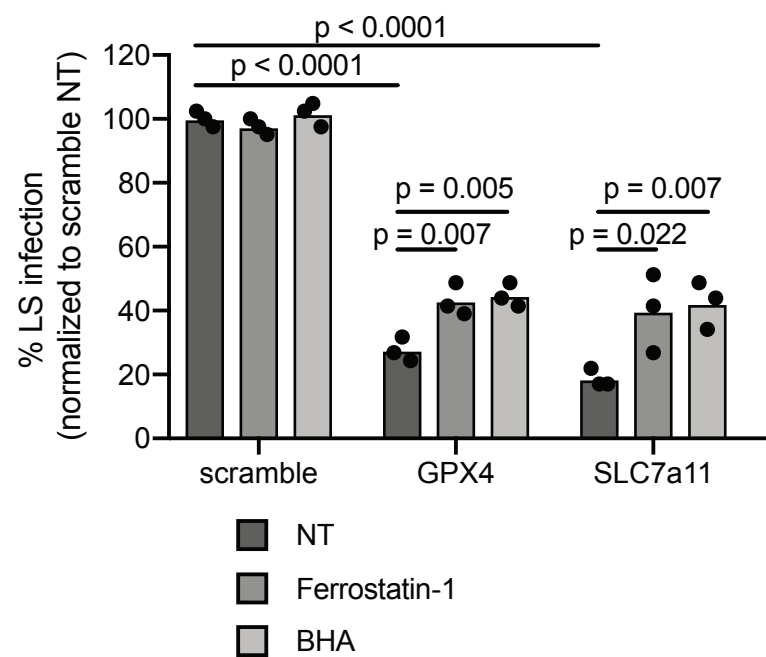

Supplement: Supplementary file 4 — Supplemental Figure 2 [file 41418_2019_338_MOESM4_ESM.pdf]

Figure S3

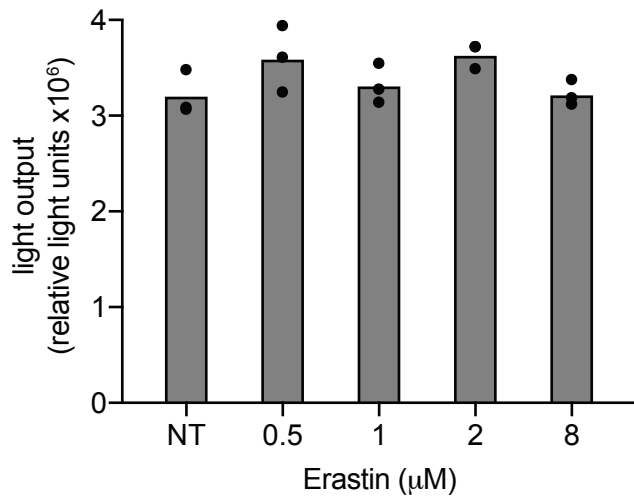

Supplement: Supplementary file 5 — Supplemental Figure 3 [file 41418_2019_338_MOESM5_ESM.pdf]

Figure S4

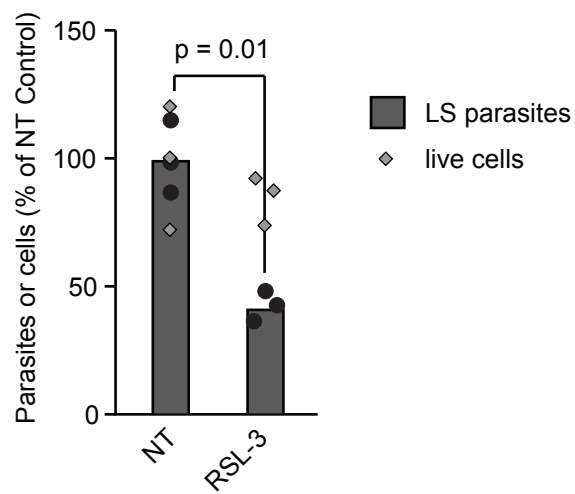

Supplement: Supplementary file 6 — Supplemental Figure 4 [file 41418_2019_338_MOESM6_ESM.pdf]

Figure S5

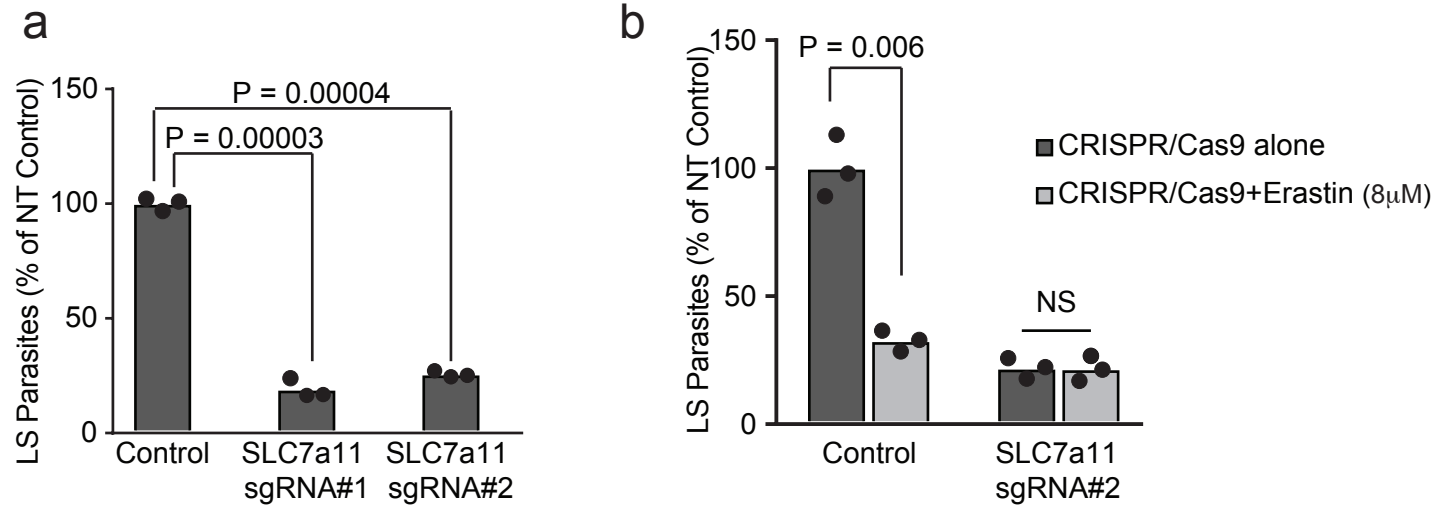

Supplement: Supplementary file 7 — Supplemental Figure 5 [file 41418_2019_338_MOESM7_ESM.pdf]
